# Supplementary material for: Transcriptomics and trans-organellar complementation reveal limited signaling of 12-cis-oxo-phytodienoic acid during early wound response in Arabidopsis
Source: Nat Commun. 2025 Jul 21;16:6684. doi: 10.1038/s41467-025-61832-9 (PMC12280005; doi:10.1038/s41467-025-61832-9)
Supplement: Supplementary file 2 — Description of Additional Supplementary Files [file 41467_2025_61832_MOESM2_ESM.pdf]

Description of Additional Supplementary files

Supplementary Dataset 1:

Genes found in the RNAseq data to be commonly differentially expressed by wounding in seedlings of Col-0, *opr2opr3* and *aos* (supporting Figure 2).

Supplementary Dataset 2:

Genes found in the RNAseq data to be differentially expressed in Col-0 compared to *opr2opr3* and *aos* mutant seedlings under wounding conditions (supporting Figure S5).

Supplementary Dataset 3:

Genes found in the RNAseq data to be differentially expressed in *opr2opr3* seedlings treated with 25 µM OPDA (*opr2opr3* + OPDA) compared to wounded *opr2opr3* seedlings producing OPDA endogenously (supporting Figure 4).
