# Supplementary material for: Transcriptomics and trans-organellar complementation reveal limited signaling of 12-cis-oxo-phytodienoic acid during early wound response in Arabidopsis
Source: Nat Commun. 2025 Jul 21;16:6684. doi: 10.1038/s41467-025-61832-9 (PMC12280005; doi:10.1038/s41467-025-61832-9)
Supplement: Supplementary file 4 — Reporting Summary [file 41467_2025_61832_MOESM4_ESM.pdf]

Corresponding author(s): Bettina Hause
Last updated by author(s): Jun 16, 2025

# Reporting Summary

Nature Portfolio wishes to improve the reproducibility of the work that we publish. This form provides structure for consistency and transparency in reporting. For further information on Nature Portfolio policies, see our Editorial Policies and the Editorial Policy Checklist.

## Statistics

For all statistical analyses, confirm that the following items are present in the figure legend, table legend, main text, or Methods section.

| n/a | Confirmed | |
|---|---|---|
| ☐ | ☒ | The exact sample size (*n*) for each experimental group/condition, given as a discrete number and unit of measurement |
| ☐ | ☒ | A statement on whether measurements were taken from distinct samples or whether the same sample was measured repeatedly |
| ☐ | ☒ | The statistical test(s) used AND whether they are one- or two-sided<br>*Only common tests should be described solely by name; describe more complex techniques in the Methods section.* |
| ☒ | ☐ | A description of all covariates tested |
| ☐ | ☒ | A description of any assumptions or corrections, such as tests of normality and adjustment for multiple comparisons |
| ☐ | ☒ | A full description of the statistical parameters including central tendency (e.g. means) or other basic estimates (e.g. regression coefficient) AND variation (e.g. standard deviation) or associated estimates of uncertainty (e.g. confidence intervals) |
| ☐ | ☒ | For null hypothesis testing, the test statistic (e.g. *F*, *t*, *r*) with confidence intervals, effect sizes, degrees of freedom and *P* value noted<br>*Give P values as exact values whenever suitable.* |
| ☒ | ☐ | For Bayesian analysis, information on the choice of priors and Markov chain Monte Carlo settings |
| ☒ | ☐ | For hierarchical and complex designs, identification of the appropriate level for tests and full reporting of outcomes |
| ☒ | ☐ | Estimates of effect sizes (e.g. Cohen's *d*, Pearson's *r*), indicating how they were calculated |

*Our web collection on statistics for biologists contains articles on many of the points above.*

## Software and code

Policy information about availability of computer code

| | |
|---|---|
| Data collection | We used ZEN software (Zeiss) to collect microscopic images. |
| Data analysis | To analyze the RNAseq-data, gene expression heatmaps, principal component analysis (PCA), gene clustering, and Gene Ontology (GO) enrichment analysis were generated using the iDEP.96 (http://bioinformatics.sdstate.edu/idep96/), iDEP 1.1 (http://bioinformatics.sdstate.edu/idep11/) and ShinyGO 0.80 (http://bioinformatics.sdstate.edu/go/) software tools. All statistical analyses were done using GraphPad Prism (www.graphpad.com). Microscopic images were processed using PHOTOSHOP 12.0.4 (Adobe Systems, http://www.adobe.com). |

For manuscripts utilizing custom algorithms or software that are central to the research but not yet described in published literature, software must be made available to editors and reviewers. We strongly encourage code deposition in a community repository (e.g. GitHub). See the Nature Portfolio guidelines for submitting code & software for further information.

# Data

Policy information about availability of data

All manuscripts must include a data availability statement. This statement should provide the following information, where applicable:

- Accession codes, unique identifiers, or web links for publicly available datasets
- A description of any restrictions on data availability
- For clinical datasets or third party data, please ensure that the statement adheres to our policy

The datasets generated and/or analyzed during this study are provided as Source Data file. Transcriptomic raw data were deposited in Sequence Read Archive (SRA) of NCBI (PRJNA1088739 [ https://www.ncbi.nlm.nih.gov/bioproject/PRJNA1088739] and PRJNA1105938 [https://www.ncbi.nlm.nih.gov/bioproject/PRJNA1105938]). All other data that support the findings of this study are available from the corresponding author upon reasonable request.

# Research involving human participants, their data, or biological material

Policy information about studies with human participants or human data. See also policy information about sex, gender (identity/presentation), and sexual orientation and race, ethnicity and racism.

| | |
|---|---|
| Reporting on sex and gender | n.a. |
| Reporting on race, ethnicity, or other socially relevant groupings | n.a. |
| Population characteristics | n.a. |
| Recruitment | n.a. |
| Ethics oversight | n.a. |

Note that full information on the approval of the study protocol must also be provided in the manuscript.

# Field-specific reporting

Please select the one below that is the best fit for your research. If you are not sure, read the appropriate sections before making your selection.

☒ Life sciences ☐ Behavioural & social sciences ☐ Ecological, evolutionary & environmental sciences

For a reference copy of the document with all sections, see nature.com/documents/nr-reporting-summary-flat.pdf

# Life sciences study design

All studies must disclose on these points even when the disclosure is negative.

| | |
|---|---|
| Sample size | For all transcript/transcriptome analyses, 3 independent samples containing 30-120 seedlings were used, for hormone analyses 3-4 samples were used. Sample size has been chosen according to the state-of-the-art in plant science. |
| Data exclusions | There were no data excluded. |
| Replication | All experiements were done three times. All attempts at replication were successful. |
| Randomization | Plants/seedlings were grown in a randomized design. |
| Blinding | All extractions (RNA, hormones) and follow-up analyses of exctracts as well as microscopical analyses of transiently or stable transformed plants were done blinded. |

# Reporting for specific materials, systems and methods

We require information from authors about some types of materials, experimental systems and methods used in many studies. Here, indicate whether each material, system or method listed is relevant to your study. If you are not sure if a list item applies to your research, read the appropriate section before selecting a response.

## Materials & experimental systems

| n/a | Involved in the study | |
|---|---|---|
| ☐ | ☒ | Antibodies |
| ☒ | ☐ | Eukaryotic cell lines |
| ☒ | ☐ | Palaeontology and archaeology |
| ☒ | ☐ | Animals and other organisms |
| ☒ | ☐ | Clinical data |
| ☒ | ☐ | Dual use research of concern |
| ☐ | ☒ | Plants |

## Methods

| n/a | Involved in the study | |
|---|---|---|
| ☒ | ☐ | ChIP-seq |
| ☒ | ☐ | Flow cytometry |
| ☒ | ☐ | MRI-based neuroimaging |

# Antibodies

| | |
|---|---|
| Antibodies used | rabbit anti-AtAOC (Stenzel et al., 2003), goat anti-rabbit IgG conjugated with alkaline phosphatase (www.sigmaaldrich.com, #AP307P). |
| Validation | Anti-AtAOC has been characterized and described by Stenzel et al. (Jasmonate biosynthesis and the allene oxide cyclase family of Arabidopsis thaliana, Plant Mol. Biol. 2003, 51:895-911). The full blots are shown in the Source Data file. |

# Dual use research of concern

Policy information about dual use research of concern

## Hazards

Could the accidental, deliberate or reckless misuse of agents or technologies generated in the work, or the application of information presented in the manuscript, pose a threat to:

| No | Yes | |
|---|---|---|
| ☒ | ☐ | Public health |
| ☒ | ☐ | National security |
| ☒ | ☐ | Crops and/or livestock |
| ☒ | ☐ | Ecosystems |
| ☒ | ☐ | Any other significant area |

## Experiments of concern

Does the work involve any of these experiments of concern:

| No | Yes | |
|---|---|---|
| ☒ | ☐ | Demonstrate how to render a vaccine ineffective |
| ☒ | ☐ | Confer resistance to therapeutically useful antibiotics or antiviral agents |
| ☒ | ☐ | Enhance the virulence of a pathogen or render a nonpathogen virulent |
| ☒ | ☐ | Increase transmissibility of a pathogen |
| ☒ | ☐ | Alter the host range of a pathogen |
| ☒ | ☐ | Enable evasion of diagnostic/detection modalities |
| ☒ | ☐ | Enable the weaponization of a biological agent or toxin |
| ☒ | ☐ | Any other potentially harmful combination of experiments and agents |

## Plants

| | |
|---|---|
| Seed stocks | Arabidopsis thaliana Col-0, aos (dde2-2), coi1-16 and coi1-30 were obtained from "The European Arabidopsis Stock Centre (NASC)". opr2-1 opr3-3 was provided by Prof. Andreas Schaller, University of Hohenheim, Germany (see Chini et al., Nature Chem. Biol. 2018) |
| Novel plant genotypes | Transgenic plants were produced using Agrobacterium tumefaciens (strain GV3101)-mediated transformation (flower dip according to Zhang et al., 2006). For all constructs, at least two independent transgenic lines harboring a single insertion were selected and multiplied to obtain T3 for further analyses. |
| Authentication | All lines used (mutants and transgenic lines) were verified by PCR using genomic DNA and primers specific for the respective mutation or the transgene. |
